# Moving from crisis response to a learning health system: Experiences from an Australian regional primary care network

**DOI:** 10.1002/lrh2.10458

**Published:** 2024-09-23

**Authors:** Bianca Forrester, Georgia Fisher, Louise A. Ellis, Andrew Giddy, Carolynn L. Smith, Yvonne Zurynski, Lena Sanci, Katherine Graham, Naomi White, Jeffrey Braithwaite

**Affiliations:** ^1^ Centre for Healthcare Resilience and Implementation Science, Australian Institute of Health Innovation Macquarie University Sydney Australia; ^2^ West Victoria Primary Health Network Geelong Australia; ^3^ University of Melbourne Melbourne Australia

**Keywords:** change management, Community of Practice, COVID‐19, crisis, crisis management, learning health system, learning healthcare systems, primary care

## Abstract

**Introduction:**

The COVID‐19 pandemic challenged primary care to rapidly innovate. In response, the Western Victorian Primary Health Network (WVPHN) developed a COVID‐19 online Community of Practice comprising general practitioners (GPs), practice nurses, pharmacists, aged care and disability workers, health administrators, public health experts, medical specialists, and consumers. This Experience Report describes our progress toward a durable organizational learning health system (LHS) model through the COVID‐19 pandemic crisis and beyond.

**Methods:**

In March 2020, we commenced weekly Community of Practice sessions, adopting the Project ECHO (Extension of Community Health Outcomes) model for a virtual information‐sharing network that aims to bring clinicians together to develop collective knowledge. Our work was underpinned by the LHS framework proposed by Menear et al. and aligned with Kotter's eight‐step change model.

**Results:**

There were four key phases in the development of our LHS: build a Community of Practice; facilitate iterative change; develop supportive organizational infrastructure; and establish a sustainable, ongoing LHS. In total, the Community of Practice supported 83 unique COVID‐19 ECHO sessions involving 3192 h of clinician participation and over 10 000 h of organizational commitment. Six larger sessions were run between March 2020 and September 2022 with 3192 attendances. New models of care and care pathways were codeveloped in sessions and network leaders contributed to the development of guidelines and policy advice. These innovations enabled WVPHN to lead the Australian state of Victoria on rates of COVID vaccine uptake and GP antiviral prescribing.

**Conclusion:**

The COVID‐19 pandemic created a sense of urgency that helped stimulate a regional primary care‐based Community of Practice and LHS. A robust theoretical framework and established change management theory supported the purposeful implementation of our LHS. Reflection on challenges and successes may provide insights to support the implementation of LHS models in other primary care settings.

## 1 INTRODUCTION

You never want a serious crisis to go to waste…it's an opportunity to do things you think you could not do before ‐ Rahm Emanuel, American politician and the former White House Chief of Staff, 2008.

The COVID‐19 pandemic was a “stress test” of health systems worldwide; it has been an exercise in complex systems adaptation and has highlighted the critical need for resilient and responsive health systems more than ever before.^1^ Across all levels of the health system, health professionals, managers, and policymakers were challenged to absorb the shock of the pandemic and rapidly design and implement new policies, technologies, and care practices.^2^ One approach to meet these challenges is to cultivate a learning health system (LHS), a concept introduced in 2007 by the United States' Institute of Medicine (now the National Academy of Medicine), wherein data from all sources are rapidly converted into knowledge that informs patient care.^3^ In this approach, incentives are aligned for high‐value care, patients and clinicians are partners in the co‐design of care, data are made widely available for stakeholder decision making, and the health workforce is engaged in continuous learning cycles of improvement.^3^, ^4^

Both conceptually and practically, mobilizing an LHS calls for healthcare organizations to be more systematic in generating and using knowledge to improve the quality of care and stimulate innovation.^5^ While the theory of LHSs has been widely discussed, studies examining the real‐world experience of implementing LHSs and the factors that facilitate their implementation in the complex and varied contexts of healthcare remain relatively scarce, although empirical work has been steadily gaining traction over the past decade.^6^, ^7^ To date, much of the literature on LHS implementation has concentrated on specific conditions, data platforms, and informatics, with less attention paid to other LHS aspects; for example, the provision of incentives, strong patient–clinician partnerships, a skilled and empowered workforce, supportive governance structures, and a receptive organizational culture.^8^, ^9^ Notably, there have been few investigations of LHSs specific to the context of primary care.^10^

In 2020, the Western Victorian Primary Health Network (WVPHN) implemented a *COVID‐19 Pandemic Response ECHO network* (referred to locally as *COVID ECHO*). An ECHO (Extension of Community Health Outcomes) model is a virtual information‐sharing network that aims to bring clinicians together to develop collective knowledge. The Project ECHO model of education was originally developed at the University of New Mexico in 2003 as a virtual program to meet the needs of clinicians living in rural and underserved locations across that US state.^11^ The Project ECHO model has since grown to a validated workforce development model that has been adopted globally across multiple sectors.^12^ Building on the initial architecture of this model, our *COVID ECHO* was established as a Community of Practice^13^ comprising management processes and learning cycles to cope with the disruptive, uncertain, and constantly changing nature of the COVID‐19 pandemic within a regional primary health system. The Community of Practice was established in March 2020, and has since grown to an embedded regional primary care led LHS that is being generalized to non‐COVID‐related health challenges. This experience report describes the implementation of this LHS model and leverages the opportunity to increase knowledge about the implementation of LHSs in primary care.

## 2 PURPOSE

This report aims to describe the challenges and successes in the implementation of an LHS model in a regional primary care setting, and to provide insights to support the implementation of LHS models in other primary care settings. Key contextual information for the Australian health system is provided in Box 1.

BOX 1 **The Australian Health System Context**

The Australian health system operates through federal‐state collaboration, where Medicare (the national universal health insurance system) provides subsidized access to essential services, and state governments oversee hospitals and public health facilities. Primary care, predominantly comprising small businesses, is federally governed and funded, with Primary Health Networks (PHNs) coordinating services to enhance local service integration and health outcomes.^14^ The WVPHN is responsible for a large rural catchment spanning 79 843 square kilometers, comprising 21 local government areas and serving ~714 000 people. While funded and governed by the federal government in the main, primary care adheres to state public health directives.

## 3 METHODS

### 3.1 Conceptual frameworks underpinning our LHS approach

**FIGURE 1:**
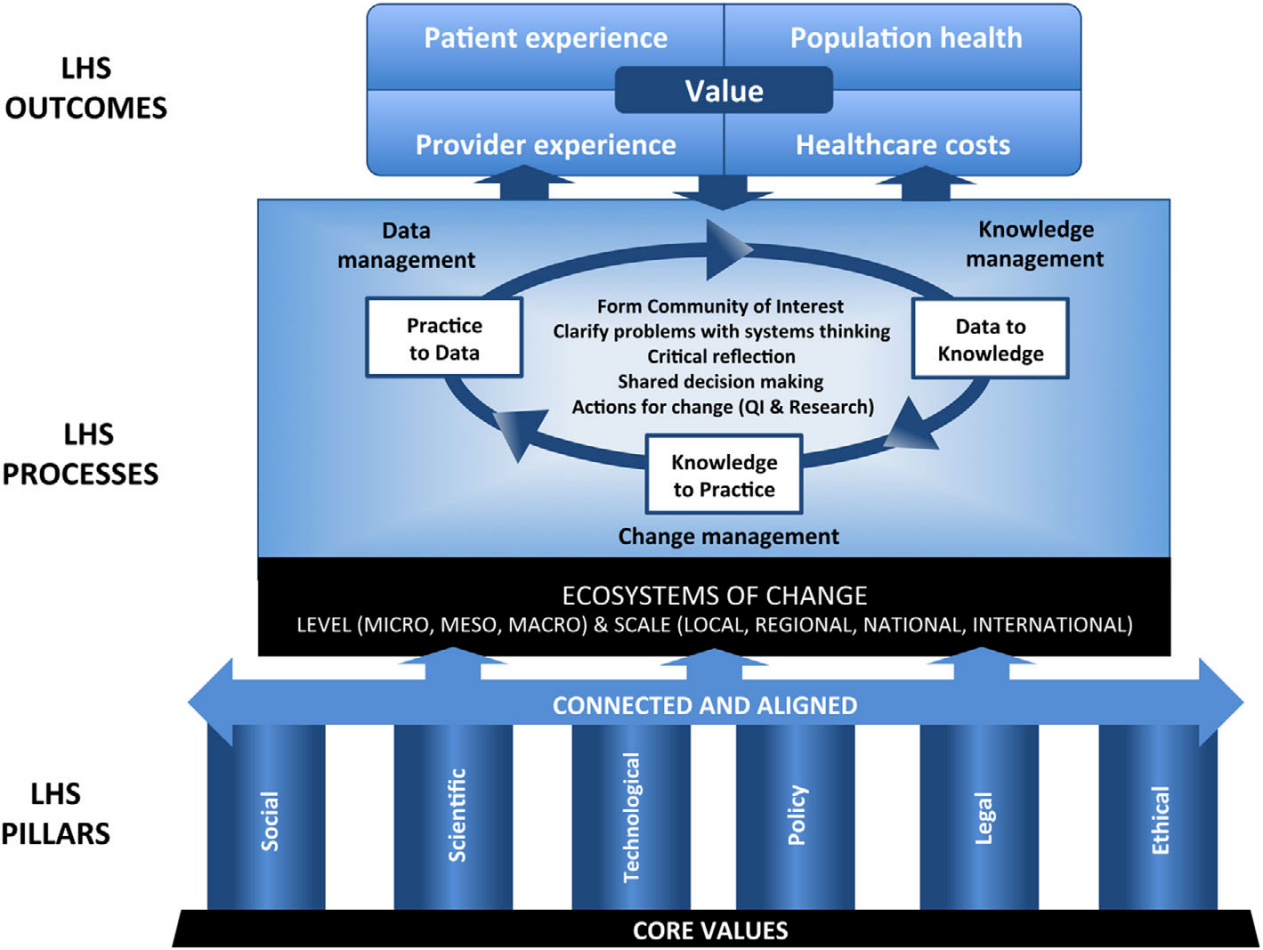
Conceptual framework for learning health systems. Reproduced with permission.^15^

Our LHS journey began in response to carefully calibrated, collective efforts to understand the COVID‐19 pandemic threat, mobilize knowledge, and manage change in response to a crisis. Our process comprised: gathering, refining, and analyzing data, building knowledge, making decisions with the best available evidence and practice, managing change, and, to complete the learning cycle, purposefully reflecting upon our impact. These steps are at the heart of an LHS as described by the Institute of Medicine.^3^ In the process of refining our LHS approach, we adopted the conceptual framework of LHS proposed by Menear and colleagues (Figure 1) to explain our work to date^15^ and aligned our work with Kotter's eight‐step change model.^16^ These eight steps and the Menear LHS framework were used to assess the success of our learning network and to guide our reflection on learnings, ideas, and possible alternate approaches.

### 3.2 Phases of development

Applying Kotter's model,^16^ the first step, “a sense of urgency,” was created for us, driven by the COVID‐19 pandemic crisis. Following on, there were four key phases in the development of our LHS:

1. Build a broad coalition and network for effective knowledge mobilization through a Community of Practice (*Kotter's Step 2: Form a Powerful Coalition*).
2. Actively facilitated an iterative approach to change management that sought to align diverse interests, establish, and communicate common goals and use systems thinking practice to identify obstacles and barriers to changing practice (*Kotter's Steps 3 and 4: Create a Vision for Change and Communicate the Vision*).
3. Develop supportive organizational infrastructure that works to remove systems barriers and collaborates to define and develop supportive care pathways across the regional system (*Kotter's Steps 5 and 6: Empower Action, Create Quick Wins*).
4. Establish a durable regional primary care led LHS, reflect upon practice, and develop a systematized LHS approach that can be applied to multiple content areas (*Kotter's Steps 7 and 8: Build on the Change and Make it Stick*).

The subsequent sections of this report discuss our efforts in each of these stages. We concentrate on analyzing the associated outcomes, successes, and challenges.

## 4 RESULTS

### 4.1 Development of a community of practice

#### 4.1.1 Phase 1: Build a broad coalition and network for effective knowledge mobilization through a Community of Practice (Kotter's Step 2)

To build a coalition, we developed communication channels with state government and regional health service specialists, public health experts, and representatives from the primary care sector. This facilitated rapid dissemination of COVID‐19 information and supported primary care providers in translating public health orders, care models, and risk mitigation strategies to diverse primary care settings in our region. Adopting the Project ECHO model,^11^ *COVID ECHO* included a diverse range of participants, fostering horizontal and vertical communication across primary, tertiary, and non‐government sectors. Participants in *COVID ECHO* included general practitioners (GPs), practice nurses, practice managers (PMs), allied health professionals, pharmacists, aged care and disability workers, health administrators, public health experts, medical specialists, and healthcare consumers based in the Western Victorian Region. The project, led by an academic GP (BF), clinical advisor (KG), and supported by a team of organizational stakeholders (including NW and AG) evolved into a dynamic coalition meeting weekly to discuss policy, scientific principles and evidence, and using systems thinking practices^17^ to enable critical reflection and translation into practice. Sessions were conducted in cohort series lasting between 8 and 12 weeks, with regular breaks scheduled as appropriate.

#### 4.1.2 Phase 2: Actively facilitate an iterative approach to change management

In Phase 2, *COVID ECHO's* responsive and iterative agenda aimed to equip participants with knowledge, skills, and attitudes to manage the impacts of COVID‐19, adopt new digital and medical technologies, and develop new care models that would enable service continuity in the face of social distancing restrictions. Each session involved a planning process to identify key issues and collect relevant data, advice and guiding principles carefully crafted to support clinician education and decision making. A core team of technical experts and local health leaders were selected for their ability to curate these data and knowledge resources. To maintain engagement, the Community of Practice leader (BF) interviewed key stakeholders across all sectors to identify key issues, common priorities, and care gaps; *a shared vision to be communicated* in the session.^16^ Thus, we ensured that we could take data and convert it into knowledge, a key process of an LHS.^15^ While each session involved clear communication of goals for the week (*Step 4: communicate the vision*), we required levels of consensus such that these goals were possible within the complex interplay of funding models, time pressures, incentives, and public health orders. Through the use of the “*all teach all learn”* ethos of the original Project ECHO, we framed primary care teams as experts in their clinical context and used facilitated case discussions to translate policy and evidence into practice, another key process of an LHS.^15^ We reviewed practice along the healthcare consumer care continuum and defined the systems gaps, weak points, barriers, and change opportunities, considering the clinician's role in changing the clinical microsystem, the health services roles in facilitating integrated care, and the PHN organization's role in addressing systemic obstacles or barriers. Through these dialogs, we developed a deep understanding of our local healthcare system, and we worked collectively toward what early systems thinking strategist, Peter Senge, has described as resolving “the creative tension between *shared vision* and our *current realit*y.”^18^ Participants were invited to critically reflect upon the clinical decision making in context and tease out the variables that enabled or constrained change.

Resistance or inherent tensions were coded as barriers or *obstacles* to change and wherever possible, ascribed to actions and behaviors by relevant actors in context. Clinicians share new practices and clinical workflows, thus converting new knowledge to practice, and sharing these innovations with peers, the next component of the LHS.^15^

#### 4.1.3 Phase 3: Develop supportive organizational infrastructure

**FIGURE 2:**
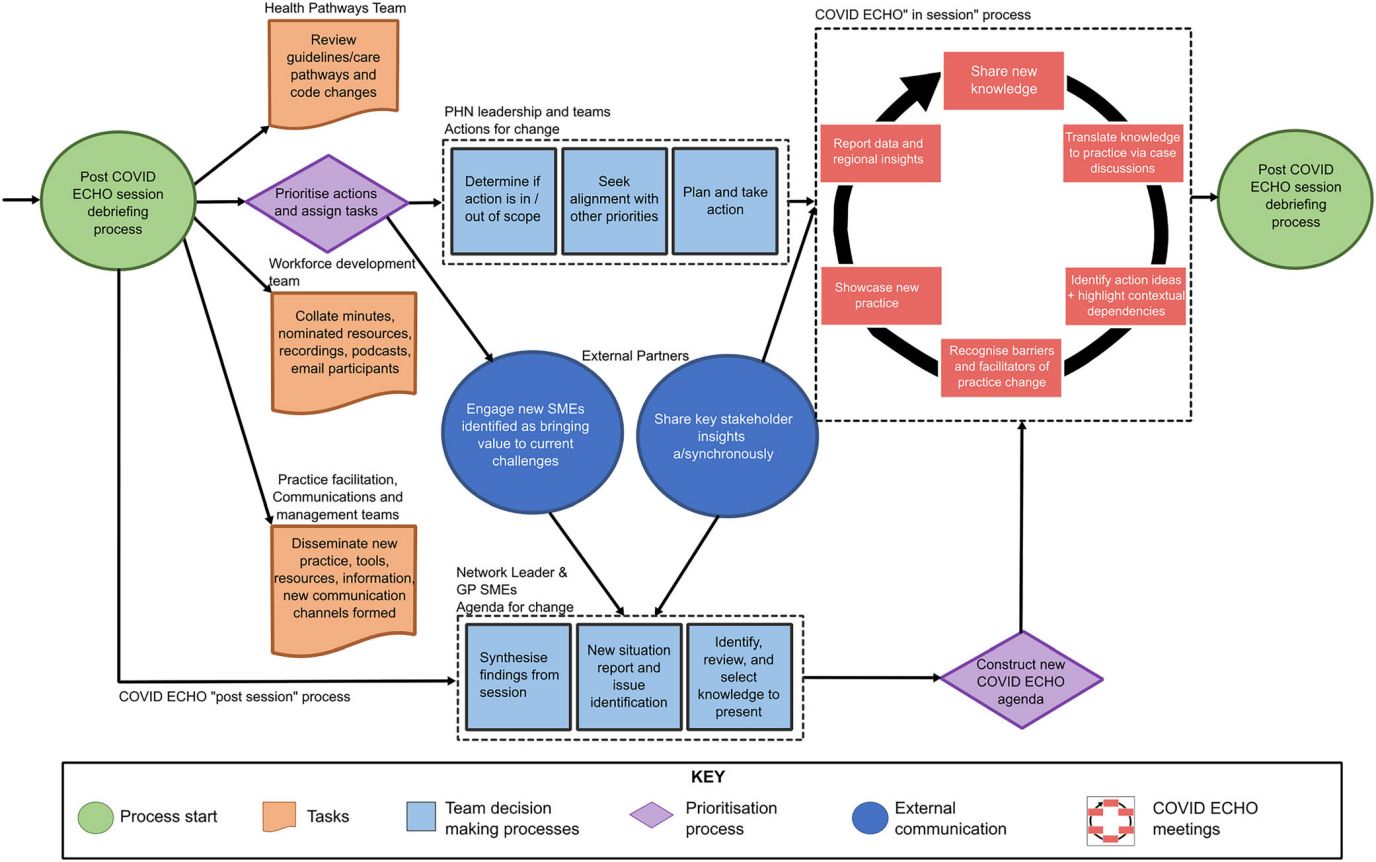
Process map of Western Victoria Primary Health Network *COVID ECHO* in Years 1 and 2 (2020 and 2021). This process map describes the WVPHN internal team processes outside the COVID ECHO sessions. External stakeholders were informally involved.

In Phase 3, a debriefing process among key leaders and organizational stakeholders followed each *ECHO* session to highlight implementation challenges for clinicians and to achieve consensus about relevant actions in each sector. There were clear expectations that relevant stakeholders would report on systems change initiatives, thereby sharing accountability for systems change. As an organizational team, we recorded and codified knowledge generated in *COVID ECHO* sessions and disseminated resources through various channels. The project's scientific GP expert (KG) was also the lead clinical editor of the state‐wide PHN COVID‐19 Health Pathways, a curated repository of guidance for primary care clinicians to support best practice care and streamlined service access.^19^ KG encoded key elements from our learning conversations into Health Pathways, ensuring they were contextually relevant across diverse clinical settings, including rural areas. Didactic presentations were recorded and shared in video, podcast, and written format after the sessions via Community of Practice mailing lists and through a web repository. Key issues were also proactively communicated across formal (e.g., health service GP liaison units) and informal (e.g., social media) networks. Over time, our *COVID ECHO* organizational team became familiar with new roles and responsibilities and our processes were routinized (see Figure 2). In the early stages, we leveraged and spread “quick wins” (Kotter's Step 6).

**FIGURE 3:**
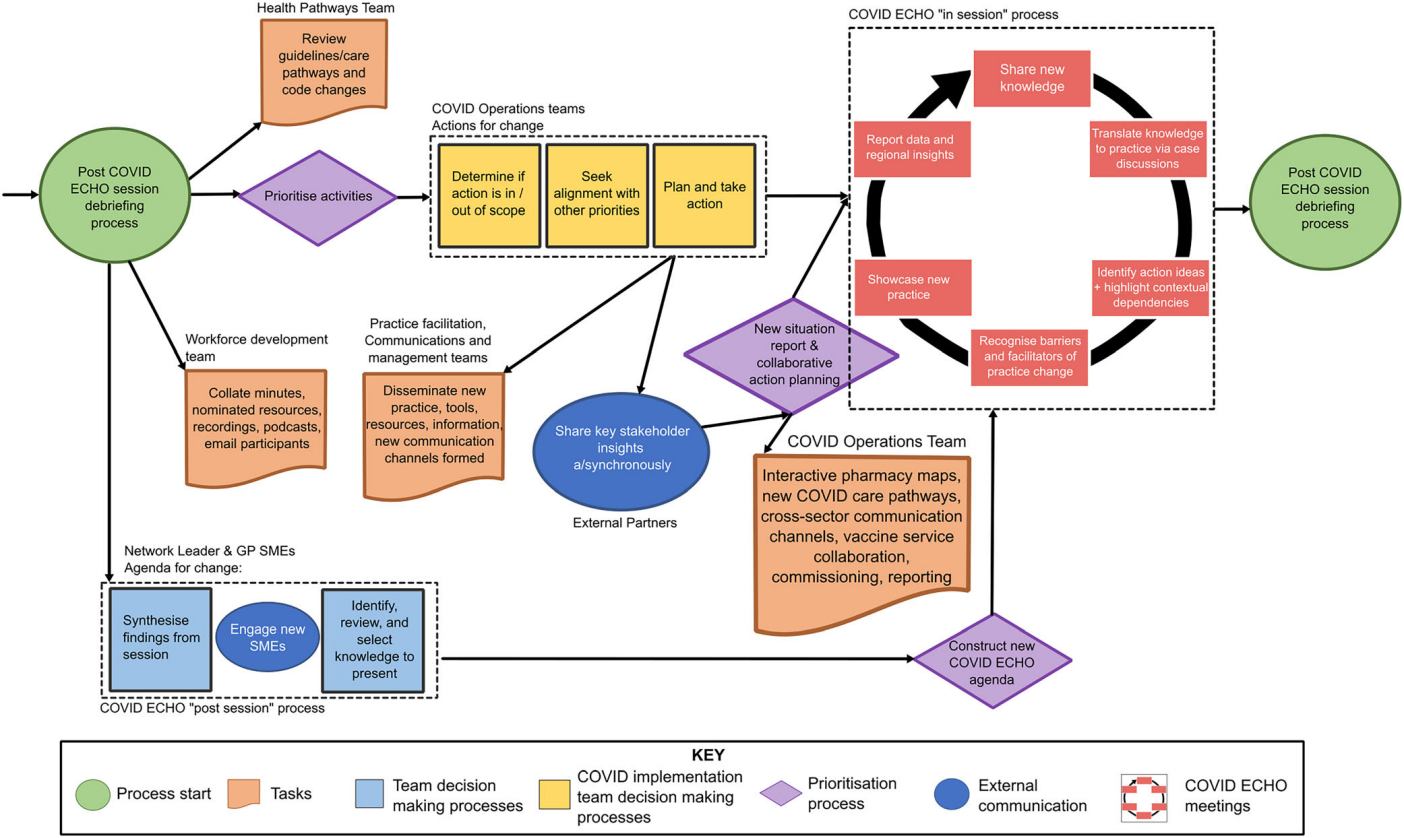
Western Victoria Primary Health Network COVID ECHO Process in Year 3 (2022) Internal teams restructured to support COVID operations and external stakeholder partnerships.

By 2022, we were ready to formalize our approach to change management. The executive team allocated COVID‐19 emergency management resources to the establishment of a COVID operational team to support the scale‐up of enabling interventions designed through *COVID ECHO*. The team leader (NW) managed the flow of data, policy, and health service‐related information through the organization, announcing policy and health services updates at *COVID ECHO*. They coordinated more formal cross‐sectoral meetings with external key stakeholders, enabling the *COVID ECHO* technical experts and facilitators to move into more strategic roles as key informants and advisors within and beyond *COVID ECHO* forums (see Figure 3). *COVID ECHO* meetings took on the function of a focus group and platform for prototyping and testing new tools, resources, and care models with scale‐up and spread supported by the COVID operational team. This investment supported the development of regional community‐based COVID care pathways in partnership with our public health units and health services.

#### 4.1.4 Phase 4: Establish a durable regional primary care led LHS

By 2022, the third year of the pandemic, it became clear that a shift from rapid response mode to a more considered pace was necessary. Our Community of Practice participants were reporting fatigue and burn‐out, but we did not want to lose the hard‐won gains of our new forum for innovation, improvement, and collaboration. In this phase, aligned with Kotter's seventh and eighth steps, we built on the change and aimed to sustain it—to help the design features endure. A facilitated workshop engaged team leaders and managers to understand the value and potential of adapting *COVID ECHO* management processes into a broader regional primary care led LHS. The network leader (BF) presented the LHS conceptual framework and invited participants to *teach back* the ways in which this model could be used by PHNs to deliver continuous improvement and innovation across various healthcare areas.

### 4.2 Outcomes

#### 4.2.1 Community of practice reach

In total, 83 unique 1‐h COVID *ECHO* sessions were run between March 2020 and September 2022 with 3192 attendances. In a region of around 700 GPs, there were on average 50 participants per session with 38 participants identified as “core” Community of Practice members (i.e., members who attended all sessions). Over half of our 220 primary care practices were represented in sessions and six larger webinar events were run to communicate key policy decisions or new technologies, or to share new practice models to a broader audience of health professionals. A total of 932 participants attended these larger events, with an average of 180 at each. Podcasts generated 927 listens, and the Health Pathways pages received 1851 views each month. WVPHN team members were involved in work which included preparing, delivering, and debriefing after each session resulting in approximately 10 000 organizational hours spent over a 3‐year period. External stakeholders provided in‐kind contributions involving similar tasks approximating over 250 h. A brief survey followed each session and was completed by a total of 71 participants over a period of 12 months. The sessions were rated as being “entirely relevant to practice” by 93% (*n* = 66) of respondents. A total of 75% (*n* = 53) rated the quality of the presentations as “excellent,” with the remaining 25% (*n* = 18) rating presentations as “good.” *COVID ECHO* participants informally reported that they were regarded as “knowledgeable by peers,” and that colleagues would ask them for information or advice about policy and practice. Key GP stakeholders in *COVID ECHO* also served as primary care advisors to health services and policymakers at times. Participants frequently expressed the timely and relevant nature of the Community of Practice and felt supported by peers through this forum.

#### 4.2.2 A sustainable community of practice

Almost 4 years after its inception, our Community of Practice is still active, and we have developed enhanced awareness of the role of primary care within a broader public health system. We have also gained a working understanding of both practice‐level and systems‐level change strategies. This progress has been driven by a responsive curriculum in public health principles and honed through case‐based discussion addressing the wide range of issues confronting Primary Care during the COVID‐19 pandemic. The content of sessions was driven by case discussion, participant questions, and interviews between sessions, ensuring sessions were highly relevant. While COVID‐19 was the central theme, sessions were diverse and spanned topics such as digital transformation, remote pediatric assessment, youth mental health, and advanced care planning. Every session involved a tug‐of‐war between “content push” and “content pull” and we worked diligently to ensure equal time dedicated to discussing how the relevant policy, epidemiology, or technology would impact and be implemented in practice. We developed what one member of our community described as a “trusted dialog” and another described as a forum “where we would learn systems change strategies that could be applied to other issues.” The interdisciplinary participation in *COVID ECHO* created greater understanding among professionals and leaders outside of the sector, of the context and challenges of primary care—that many factors in addition to knowledge and skills determine whether or not change is adopted in practice.^20^

Frequent communication and reflection between sessions enabled this responsive curriculum, and the network's agenda was kept deliberately flexible to allow the prioritization of emerging issues. Balancing the needs and expectations of multiple providers proved challenging, requiring careful communication and transparent decision‐making processes to manage potential risks to new relationships. To achieve balance and manage the risks, communication processes needed to be adapted and formalized. This was done by creating and allocating resources to new roles that were dedicated to managing relationships and communication across both the network and the primary and tertiary care sectors.

Despite proactive engagement strategies, participation rates fluctuated over the years, prompting the leadership team to reflect on the costs and benefits of maintaining the platform. We took regular network‐wide breaks and frequently surveyed participants about their willingness to continue meeting. There was a strong demand for ongoing sessions, with participants acknowledging the benefits of peer support and expressing eagerness to continue exploring broader Primary Care and Public Health issues through this forum.

#### 4.2.3 New models of care, guidelines, and care pathways

**FIGURE 4:**
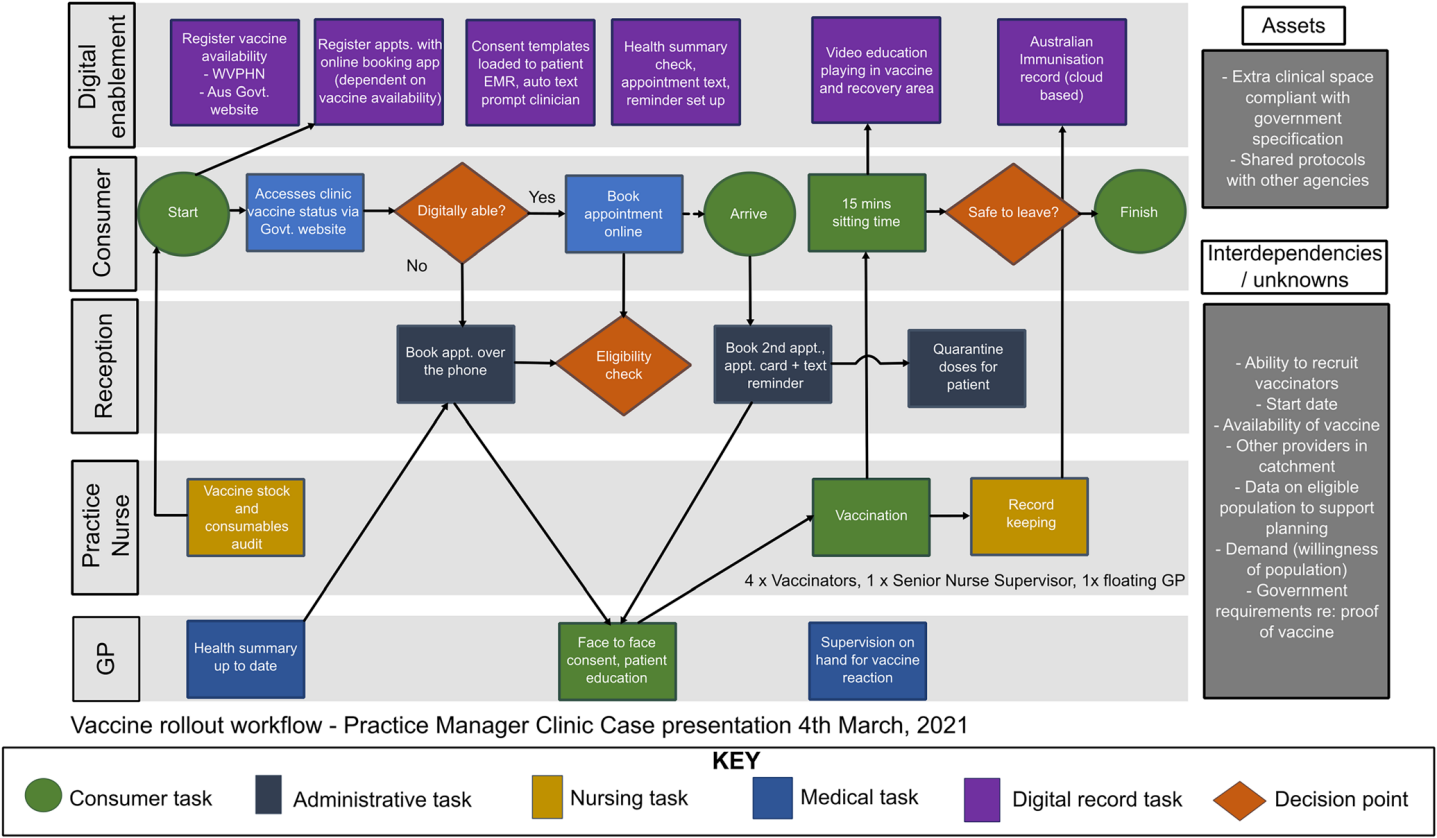
Example of the model of care design work representing the tasks of consumers and primary care teams. Arrows representing integration elements and dependencies requiring practice coordination.

*COVID ECHO* participants developed new models of care for clinic triage, telehealth, vaccination, anti‐viral prescribing, and COVID care (see Figure 4). They received advice from colleagues during sessions about implementing these models in their diverse settings.

A challenge for us as rural and regional clinicians was adapting guidance to the diverse contexts and issues with accessibility that characterize rural primary care. Case‐based discussions provided valuable insights into the way that new guidance was interpreted and managed by rural clinicians in rural contexts. As a lead GP stakeholder and lead editor for the state‐wide COVID‐19 Health Pathways, KG made improvements or wrote new guidance in response to technical issues raised in sessions.

Another challenge encountered was the fragmented nature of Australian primary and tertiary care systems, compounded by distinct governance models. To improve integration, the WVPHN team initiated regular discussions with key stakeholders in primary care, public health units, and health services outside of *COVID ECHO* sessions to ensure that local consumer care pathways were collaboratively designed. During sessions we used case‐based discussions to test consumer care journeys from community settings through health services, addressing both medical and social care needs. This strategy proved valuable, though it highlighted an even greater need for information and support for capacity building than tertiary services could provide at that time. GPs with experience in both settings became our greatest allies, and we encouraged them to adopt enhanced roles as peer educators. Several core GP members of the *COVID ECHO* Community of Practice proactively sought employment in health services and public health units to gain skills and knowledge in hospital‐based remote patient care management. These GPs shared their insights and skills in sessions to bolster GP confidence in remote patient monitoring, preparing for the eventual divestment by health services.

We also learned the importance of monitoring diverse data and information sources and listening for unintended consequences of our initiatives. One example of this was early in the COVID‐19 vaccine rollout, where we identified primary care clinics with varying caseloads and developed a cooperative approach to balancing vaccine supply and demand across neighboring clinics. We discovered that divesting from activities is as important as initiating them, with an instance of vaccine oversupply indicating the need for improved monitoring and coordination efforts.

#### 4.2.4 Health system and population health outcomes

In addition to building infrastructure for knowledge transfer (*COVID ECHO*), establishing cross‐sectoral networks, developing integrated care pathways, and creating durable guidance (Health Pathways for COVID), the WVPHN regional catchment achieved the highest second‐dose COVID‐19 vaccination rates^21^ in Australia and demonstrated the highest rates of anti‐viral prescribing in the state of Victoria.^22^

#### 4.2.5 A durable LHS model for the region

The COVID‐19 pandemic created an environment of disruption, uncertainty and rapid change that required swift yet careful navigation to maintain safe and high‐quality health services. While this was largely achieved, common issues included missing information, poor communication, misaligned goals, and duplication of efforts. The learning cycles were effective in overcoming many of these issues due to the iterative and collaborative nature of the practices involved. There was clear willingness, from clinicians, health service leads, and PHN administrators, to continue to collaborate toward shared goals and to identify and divert resources to mutually beneficial systemic solutions, all of which strengthened the performance of our LHS. In late 2022, the WVPHN executive leadership team and board formally recognized our socio‐scientific LHS approach as a viable model for innovation, knowledge translation, and workforce capacity building to drive improvement. This improvement worked toward the quintuple aim of healthcare; improving population health, enhancing care experience, supporting providers, reducing cost, and addressing inequity,^23^, ^24^ which was a strategic priority of WVPHN.^25^ We were authorized to continue applying the LHS approach to other health issues in 2023. Leadership roles and governance structures were established, including an executive sponsor (AG), network leader (BF), Community of Practice Coordinator, steering committee, and workforce development team support. Team managers and leaders expressed a willingness to partner more broadly across the organization on LHS projects addressing other health topics and to support the future development of our LHS model.

## 5 DISCUSSION

### 5.1 A Regional primary care led LHS

Through *COVID ECHO* and cross‐sectoral activities, we developed infrastructure supporting knowledge facilitation and change management during the pandemic. Since early 2020, we have leveraged over 100 learning conversations and embedded a culture of continuous learning and improvement, addressing “*complexities in public health; the ‘wicked’ public health problem, the messy policy and practice environment, and the dynamic nature of an open and heterogeneous primary care system*.”^26^ Culture change is an asset described by Menear and colleagues as “one of the most challenging tasks of LHS implementation.”^15^ We developed continuous learning routines, an enabled culture and a supportive organizational infrastructure to share data, and generate and disseminate new knowledge and practice. Each time we moved around a learning cycle we reflected upon, refined, and improved our processes, accelerating implementation.^15^ Through an active process of identifying and working toward shared goals, we strengthened our relationships and partnering capabilities, which were key processes and social foundations for our emerging LHS.^15^ The novelty, speed, and complexity of our response meant that it was not possible to establish how our region would have fared in the absence of our learning network. In the future, we plan to undertake a formal evaluation of our efforts which could support a comparison of our experience with that of other regions.

### 5.2 Accelerating knowledge management

In Community of Practice sessions, participants collaboratively interpreted changing data and policy advice, generating new knowledge for primary care clinical practice and participants' workflows. Members were trusted and regarded as knowledgeable by peers and innovative repertoires and practices were diffused through professional and social networks after sessions. These social learning processes are regarded as key to knowledge mobilization and management^13^, ^15^, ^26^ and contribute to the success of our LHS. Over the course of the pandemic, we formalized our knowledge management processes, actively mobilizing new knowledge through new and existing dissemination channels, such as Health Pathways, weekly newsletters, and web repositories. Initially, our activities were rushed and reactive with limited time between policy announcements to expected implementation. Over time and by adopting systems thinking techniques we developed the ability to critically reflect on the changing environment, recognize patterns, and predict clinician responses enabling us to better anticipate and plan communications, workforce development activities, and support needs. These staged and conscious approaches to capturing emergent new practices and actively managing knowledge diffusion, dissemination, and implementation are described as key processes in knowledge translation in a complex system.^27^

### 5.3 Accelerating change management

*COVID ECHO* emerged as a trusted local forum for resolving policy, evidence, and implementation challenges in the dynamic pandemic environment. While developed for corporate organizational settings, Kotter's eight‐step change strategies^16^ resonated with the private practice model of primary care. Through deliberative dialog, *COVID ECHO* participants made trade‐offs between policy directives, patient‐centered care, occupational health and safety, and financial sustainability, striving to address barriers and enablers to change in practice. Bringing clinician literacy about factors that constrain or create the potential for change to the fore enabled rich and insightful discussions about the nature of clinical decision making in a complex context. Key themes emerged as we discussed care across three levels of the health ecosystem: *me with my patient*, *me within my practice*, and *we within the system*. Through a process of turning tacit knowledge into an explicit understanding of change at different levels, we developed strategies and identified relevant actors who could influence implementation efforts at different levels of the system. Our high rates of vaccination and antiviral prescribing were attributed to collaborative planning and action across multiple parts of the system and were the result of these multi‐faceted interventions,^22^ many of which were developed and tested through the *COVID ECHO* sessions. Facilitating sense making about evidence in context, mobilizing opinion leaders, creating social bridges and amplifying emergent practices are key processes in successful implementation efforts.^27^

### 5.4 Accelerating data management

In the early stages of the pandemic, primary care clinicians recognized that occupational health and safety decisions were dependent on timely access to local epidemiological data. Lack of access to this data was a challenge in the early stages of the pandemic until the necessary infrastructure and communication channels were developed. Another challenge was the limited access to data for measuring the impact of our primary care interventions. Other health systems have effectively leveraged data to prepare for, and then respond to, pandemic events.^28^ For example, the Randomized Embedded Multifactorial Adaptive Platform for Community Acquired Pneumonia (REMAP‐CAP) trial integrated research into the routine care of hospitalized patients and then adapted these infrastructures for disease tracking and outcomes monitoring during COVID‐19.^29^ Notably, most examples of electronic health records (EHRs) used to prepare and respond to pandemics have occurred in larger health systems, or hospital networks.^28^ These systems and networks have greater resources, informatics capabilities, and widespread use of EHRs, making them well‐suited for data utilization. Future work should focus on how to build similar data capabilities into the more heterogeneous landscape of primary care provision, particularly in regional areas.

Additionally, it would have been interesting to observe the potential impact that high COVID‐19 vaccination rates and antiviral prescribing in our region had on the provision of other preventative health services such as influenza vaccination, cancer screening, and chronic diseases management. Enhanced surveillance of these episodes of care, potentially through monitoring EHR‐derived indicators and linked data sets, could provide valuable information for assessing the impact of future wide‐scale interventions.^30^, ^31^ Governance constraints over data sharing limited our access to health services and public health data that could inform us about the value of our efforts in preventing hospitalizations and death. In the future, data linkage and sharing between primary care and health services, supported by data governance would enable primary care to participate in celebrating the success of their efforts and support investment in preventative activities in practice.

### 5.5 Sustaining change

As the pressure of the COVID‐19 pandemic eased and the demand for continual updates and new knowledge decreased, we recognized the impact of our collaborative and co‐ordinated LHS approach and aimed to sustain both the network and the organizational processes and practices developed during the COVID‐10 pandemic. Thus, we made the deliberate decision to rebrand the network as the “*Population Health ECHO Network*” instead of simply dissolving the Community of Practice. As leaders of our Community of Practice, we initiated a collaborative design approach to developing a robust LHS model. Organizational teams assessed assets and enablers related to data, knowledge, and change management and reflected on barriers to the implementation of our new LHS process model. We recognized the opportunities that we could leverage through our digital health and data analytic team capabilities and our progress benefited from access to de‐identified electronic medical record (EMR) data and our regional data warehouse. As we went about reviewing our data governance and sharing agreements, we considered ways in which we could securely and effectively work with our local public health units (PHU) toward the shared goals of better regional insights and collaborations. We initiated “safe‐to‐fail” data extraction experiments that brought our PHN and PHU teams together again, leading to organizational learning and improvement as we partnered to solve problems across the system.^32^ Embracing a newfound interest in data and epidemiology, we developed activities to assist Community of Practice clinicians explore EMR data extraction tools in planning and conducting Quality Improvement (QI) initiatives. However, a lack of incentives to participate in research or QI activities has been identified as a barrier to engagement in LHSs in other parts of the world.^33^ Leveraging the strategic window that emerged, we supported clinicians as a component of learning in meeting professional development requirements^34^ through the development of an accredited QI activity, using routinely collected data for outcomes measurement.^35^

Of course, short‐term practice changes and trade‐offs adopted by workers in the face of a crisis may not always be sustained, or even lead to burnout, over the longer term.^2^ We recognized that participants valued the peer support that the Community of Practice offered and understood the importance of maintaining GP engagement beyond the immediate, crisis‐driven circumstances. As healthcare workers moved into a recovery phase of the pandemic, we refocused our theory‐of‐change toward what Kotter has characterized as a “*melting iceberg*”; that is, by continuously nurturing genuine “buy‐in” and commitment rather than relying on continuous pressure.^36^ We evaluated sessions and elicited feedback on members' motivations for participating and we identified professional development needs that we could meet through the ongoing Community of Practice activities, aiming to incentivize participation by aligning with new professional development requirements. In the longer term, ensuring sustainability will depend on our program “staying the course,” which could be achieved via strategic alignment with primary care reform incentives, and with state, federal, and local priorities. Broadly, this would involve creating an adaptive regional LHS model and an associated program of work that not only meets local needs but also delivers on the broader strategic aims of the WVPHN and the quintuple aim of healthcare.^14^, ^24^

### 5.6 Recommendations

Recognizing the interdependence of the primary, secondary, and tertiary health systems, sustaining an LHS in primary care depends on mobilizing a wide range of willing stakeholders and encouraging their engagement across time in the context of shared priorities and federal and state bilateral agreements. Investment in LHS design, implementation, and evaluation is crucial for navigating the complexities of the changing healthcare and digital landscape and the uncertain future of primary care reform, not just in response to a crisis. State and federal bilateral agreements, shared priorities and joint commissioning create the necessary policy levers to sustain this adaptive change mechanism. While digital transformation has accelerated virtually at an exponential pace through the course of the pandemic, our initial interrogation of our electronic medical record extraction tools and data analytics capabilities demonstrated that more investment and resourcing is required if we are to use routinely collected data from primary care to supplement public health surveillance.^31^, ^37^ Furthermore, demonstrating value in health outcomes and quality indicators will require not only shared governance arrangements between state and federal policy and legal entities but also significant investments in the workforce of the future.^38^ Continuous capability building and providing new incentives will drive the next iteration of the transformation in healthcare that is clearly needed.

## 6 CONCLUSION

To be prepared for the next crisis, we must learn from the last and project those lessons into the future. Our regional primary care led LHS was predicated on trust, collaboration, and effective use of data, and catalyzed by a sense of urgency created by the pandemic crisis. It was accelerated by the alignment of interests across primary and tertiary sectors toward the strategic goals of better patient outcomes, provider experience, population health, and equity.

## AUTHOR CONTRIBUTIONS

Bianca Forrester, Andrew Giddy, Katherine Graham, Naomi White, and Lena Sanci conceptualized the design and methods. Bianca Forrester and Georgia Fisher drafted the manuscript. Andrew Giddy, Katherine Graham, Louise A. Ellis, Carolynn L. Smith, and Yvonne Zurynski contributed to the drafting and critical review of the manuscript. Lena Sanci, Louise A. Ellis, and Jeffrey Braithwaite provided supervision. All authors contributed to its revisions and take responsibility for its contents.

## CONFLICT OF INTEREST STATEMENT

The authors have no conflicts of interest to disclose.
